# Impact of methyl Jasmonate on blueberry ripening fruits: assessment of cell wall thermal stability, nutritional parameters and antioxidant enzymatic activity

**DOI:** 10.3389/fpls.2025.1550131

**Published:** 2025-03-27

**Authors:** Carlos Vasquez-Rojas, Marcelo Muñoz-Vera, Sebastián Flores, Mauricio Betancourt, Ricardo I. Castro, Patricio Ramos, Daniel Laporte, Carolina Parra-Palma, Luis Morales-Quintana

**Affiliations:** ^1^ Multidisciplinary Agroindustry Research Laboratory, Instituto de Ciencias Biomédicas, Facultad Ciencias de la Salud, Universidad Autónoma de Chile, Talca, Chile; ^2^ Programa de Doctorado en Ciencias Biomédicas, Facultad Ciencias de la Salud, Universidad Autónoma de Chile, Talca, Chile; ^3^ Multidisciplinary Agroindustry Research Laboratory, Instituto de Ciencias Químicas Aplicadas, Facultad de Arquitectura, Construcción y Medio Ambiente, Universidad Autónoma de Chile, Talca, Chile; ^4^ Plant Microorganism Interaction Laboratory, Instituto de Ciencias Biológicas, Universidad de Talca, Talca, Chile; ^5^ Carrera de Química y Farmacia, Facultad Ciencias de la Salud, Universidad Autónoma de Chile, Talca, Chile; ^6^ Vicerrectoría de Investigación y Postgrado, Universidad Católica del Maule, Talca, Chile; ^7^ Laboratory of Plant Physiology and Molecular Biology, Instituto de Ciencias Biomédicas, Facultad Ciencias de la Salud, Universidad Autónoma de Chile, Talca, Chile

**Keywords:** antioxidant capacity, blueberry fruit ripening, cell wall, hormonal treatment, methyl jasmonate, phenols

## Abstract

**Introduction:**

The blueberry (Vaccinium spp.), recognized as one of the most significant horticultural crops globally, is valued for its rich bioactive compounds. In this study, we examine the effects of methyl jasmonate (MeJA) on blueberry, focusing on cell wall composition, nutritional properties, and antioxidant enzyme activity across two seasons (2022–2023). The objective is to evaluate the impact of MeJA treatments on fruit ripening dynamics and quality attributes.

**Methodology:**

Blueberry plants were treated with single (T1) and double (T2) MeJA applications. Thermogravimetric analysis (TGA) and differential scanning calorimetry (DSC) were used to assess thermal degradation patterns of cell wall polymers. Biochemical evaluations included phenolic content, antioxidant capacity (DPPH and FRAP assays), and anthocyanin accumulation during ripening. Enzymatic antioxidant activities (APX, CAT, SOD, and POD) were also analyzed to determine oxidative stress responses.

**Results and discussion:**

Thermal degradation analysis revealed that green-stage fruits exhibited higher thermal stability than ripe fruits, with variations in pink-stage behavior between seasons. Biochemical assessments indicated a progressive decline in phenolic content and antioxidant capacity during ripening, whereas anthocyanin accumulation peaked in the blue stage, enhancing pigmentation. MeJA treatments significantly influenced antioxidant enzyme activity: T1 maximized APX, CAT, and SOD activities, while T2 amplified POD activity, contributing to oxidative stress tolerance and improved fruit quality. Furthermore, the modulation of hemicellulose fractions in TGA profiles suggests that MeJA helps maintain cell wall integrity, potentially reducing fruit softening during storage.

**Conclusion:**

These findings indicate that MeJA enhances fruit resilience during ripening while preserving key biochemical properties critical for postharvest management. The observed improvements in antioxidant capacity, enzymatic activity, and cell wall stability suggest that MeJA could be a valuable tool for optimizing postharvest handling, extending shelf life, and enhancing the marketability of blueberries. This work provides a preliminary framework for integrating MeJA into sustainable horticultural practices to meet consumer demand for high-quality functional fruits.

## 1 Introduction

Blueberries (*Vaccinium corymbosum*) are recognized as one of the top five healthy fruits recommended by the Food and Agriculture Organization of the United Nations (Huang et al., 2018). They are renowned for their rich nutritive composition, boasting a high concentration of biologically active components, including anthocyanins, polyphenols, and flavonoids. Blueberries fruits are among the most nutrient-dense berries, abundant in fiber, vitamins, and antioxidant compounds (Kalt et al., 2020; Neto, 2007; Silva et al., 2020; Zafra-Stone et al., 2007). These antioxidants, part of the broad group of flavonoids and phenolic acids, possess functional properties that contribute to reducing the risk of heart disease, diabetes, and neurodegenerative disorders (Kalt et al., 2020; Neto, 2007; Silva et al., 2020; Zafra-Stone et al., 2007). The chemical composition of blueberries varies significantly depending on factors such as cultivar, variety, growing location, environmental conditions, plant nutrition, ripeness stage, harvest time, and storage conditions (Bujor et al., 2016; Lohachoompol et al., 2004; Skrovankova et al., 2015). These variables influence the content of individual components, thereby impacting the corresponding antioxidant profiles. Thus, finding solutions that allow improving the antioxidant profile of blueberries in post-harvest seems to be a good idea, and in this sense, various authors have described that Methyl Jasmonate (MeJA) could be a good candidate. Understanding these variations is crucial for optimizing the health benefits and nutritional value of blueberries (Bujor et al., 2016; Skrovankova et al., 2015). But it is necessary to understand its mode of action and the time and number of applications.

Methyl jasmonate (MeJA) is considered an important plant hormone that mediates intra- and inter-plant communications, modulating plant defense responses, including antioxidant systems (Wasternack, 2007; Wasternack and Hause, 2013). Thus, MeJA can significantly influence the ripening process of blueberries, enhancing fruit quality, delaying decay, and improving resistance to environmental stressors (Huang et al., 2015). In different species, such as barley and cauliflower, MeJA application has been shown to enhance enzymatic antioxidant activity and reduce cellular membrane damage caused by water deficiency (Wu et al., 2012). For example, MeJA application can regulate the production of terpenes, as lactones and carotenoids, and esters (Li et al., 2006; Cai et al., 2020); other example, in MeJA postharvest treatment of olives, not only enhanced the concentration of phenolic acids, but also decreased the levels of unhealthy saturated fatty acids and increased the content of unsaturated fatty acids: such as oleic, linoleic and linolenic acids (Flores et al., 2017). Particularly, in blueberry studies have shown that exogenous application of MeJA can accelerate ripening by increasing anthocyanin biosynthesis, phenols and flavonoids accumulations, which enhances the fruit’s color and antioxidant properties in the fruits during postharvest (Huang et al., 2015; Balbontín et al., 2025; Parra-Palma et al., 2025).

For this reason, it has been suggested that the application of MeJA reduces the activity of enzymes that hydrolyze glycosidic linkages among cell wall components, inducing cell wall softening in fruits. Consequently, the MeJA application improves fruit firmness and resistance to mechanical damage, reducing in an indirect manner the microbial attacks (Balbontín et al., 2025; Bari and Jones, 2009). These combined effects make MeJA a valuable tool in postharvest treatments to improve the marketability and shelf life of blueberries.

In other hand, the softening process is a crucial factor determining the quality and storage life of many fruits. The intricate interaction of elements and mechanisms, in plant cell walls underscores their changing nature and crucial significance in the growth and functioning of plants. Additionally, very little is known about cell wall remodeling during fruit development and the softening process in blueberry fruits. Although the processes involved in polysaccharide solubilization and depolymerization have been extensively studied using various techniques across a wide range of fresh fruits, these processes can exhibit considerable variability in both extent and timing among different species and even among cultivars of the same species, resulting in differing rates of fruit softening. Despite the extensive research in this area, the application of a thermal approach to study the degradation of cell wall polymers remains relatively novel. The impact of MeJA on the quality of blueberry fruits and the components of the fruit’s cell wall, following differential applications of MeJA directly on the plant in blueberry production farms, has yet to be explored (Castro et al., 2021a; Jara et al., 2019).

Therefore, the current investigation focuses on exploring the principal quality traits of blueberry fruits obtained from plants that were treated with exogenous MeJA at two different times. Additionally, we evaluated the degradation and thermal characteristics of the cell wall in blueberry fruits ‘O’neal’, one of the most cultivated in Chile (https://www.odepa.gob.cl/), and the effect of MeJA on the cell wall components of blueberries after exogenous application in commercial blueberry plants, using thermogravimetric analysis (TGA) and differential scanning calorimetry (DSC), in conjunction with various other relevant parameters related to fruit quality.

## 2 Materials and methods

### 2.1 Plant material

**Figure 1:**
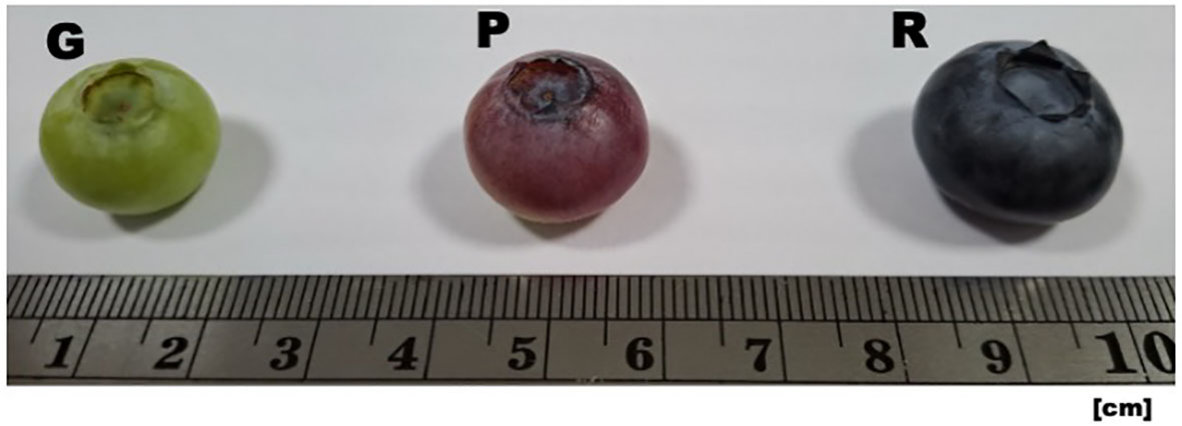
Different ripening stages of Blueberry fruits: G green fruit; P pink fruit or 50% Ripe fruit; and R ripe fruit, which correspond to 20 to 25 days post-anthesis, 35 and 45 days post-anthesis, and 50 and 60 days post-anthesis, respectively.

Fruits harvested from the *V. corymbosum* ‘O´Neal’ were obtained from 25-year-old shrubs cultivated in a commercial orchard located in Cauquenes, Maule Region, Chile (latitude 35°58′02′′ S; longitude 72°18′56′′ W) in two productive season, December 2022 and December of 2023, from trees grown in fields of soils come from granitic and metamorphic rocks, highly clayey. The transportation of the fruits was carried out in containers with dry ice, ensuring cold storage conditions within a temperature range of 4 to 8°C until their arrival at the Multidisciplinary Agroindustry Research Laboratory of the Universidad Autónoma de Chile. Upon arrival, the fruits were categorized into three different stages of growth based on criteria such as weight and color, following the classification system proposed by (Gupta et al., 2015), which included green (G), pink (P), and ripe (R) stages, which correspond to 20 to 25 days post-anthesis, 35 and 45 days post-anthesis, and 50 and 60 days post-anthesis, respectively (**Figure 1**). To conduct the analyses, nine different plants per treatment MeJA (T1 and T2), and controls (C) were utilized, more other different nine plants to collect development state fruit were utilized to collect fruits representing each of the three ripening stages, with around thirty berries sampled from each stage and treated fruit per plant, and the blueberry fruits were collected from various parts of the tree, including the upper and lower sections, the central area, as well as from locations exposed to sunlight and those in shaded areas.

### 2.2 MeJA exogenous treatment

For MeJA treatments, nine plants organized in different random locations of the commercial orchard, were treated sprinkling with a water solution containing 0.1 mM MeJA and 0.05% Tween-20. In the case of nine control plants, a solution of distilled water and 0.05% Tween-20 was sprayed. The MeJA concentration (0.1 mM MeJA) was selected based on previous studies conducted by (Cocetta et al., 2015). The solution (100 mL per bush) was applied to the above-ground parts of the blueberry bushes. This was done either as a single when the berries began to develop color (turning point), or double application (with the second application was 7 days after the first application). Then, fruits from treated plants were harvested at the ripe (R) stage on the same day, one hour from the last MeJA application.

### 2.3 Physiological parameters evaluations

Firstly, the fruit size was evaluated using a digital caliper to measure the equatorial diameters. The means and standard deviations of thirty measurements were provided per treatment and controls. Soluble solids content in the entire fruit was measured using a portable refractometer (0–32°Brix). One millilitre of homogenised pulp, obtained by grinding five berries, was used for the measurement. The analysis was performed three times for each fruit stage, and the median and average deviation for the replicates were recorded and expressed as °Brix. Colour measurements were taken from ten random were obtained from the pool, using a Nix Pro 2 Color Sensor (Nix Sensor Ltd. Hamilton, ON, Canada), and the results were expressed using the Hunter scale (L*, a*, b*). Nix Color was used to generate a schematic representation of the CIELAB color system. Color changes were expressed as E* using the following formula (see Equation 1):


(1) $$\Delta \text{E}*={\left(\text{Δ}{\text{L}}^{2}+\text{Δ}{\text{a}}^{2}+\text{Δ}{\text{b}}^{2}\right)}^{1/2}$$

where ΔL, Δa*, and Δb* represent the differences between the initial and final values for color change determination (Castro et al., 2023; Parra-Palma et al., 2024) (**Supplementary Table S1**).

### 2.4 Thermogravimetric analysis and differential scanning calorimetry

Thermogravimetric analysis (TGA) was performed in order to assess the stability of *V. corymbosum* fruits, in accordance with the methodology outlined by Castro et al. (Castro et al., 2021b). Following a drying period of 48 hours at 80°C, each sample cohort underwent homogenization through employment of a mortar. Subsequently, 5 mg of desiccated material from each sample group was subjected to scrutiny utilizing a Discovery SDT-Q650 simultaneous DSC-TGA instrument (manufactured by TA Instruments, based in New Castle, DE, USA). The specimens were subjected to a temperature range spanning from 50°C to 500°C, with a consistent escalation rate of 5°C/min, all conducted under a nitrogen gas stream maintaining a flow rate of 50 mL/min.

Differential scanning calorimetry (DSC) was conducted utilizing a Discovery SDT-Q650 simultaneous DSC-TGA instrument from TA Instruments, located in New Castle, DE, USA. The specimens were placed in an Al_2_O_3_ crucible and subjected to analysis within a temperature range spanning from room temperature (25°C) up to 500°C. Prior to the commencement of heating at a rate of 5°C per minute, the samples were allowed to stabilize at 25°C for one minute. The calibration of the equipment was carried out utilizing sapphire as the benchmark material, in accordance with the guidelines provided by the manufacturer. Each evaluation involved the utilization of 15 to 20 mg of desiccated fruit specimens. The computation of parameters such as transition enthalpy (H, expressed in J g^−1^), onset temperature (T_o_), peak temperature (T_p_), and conclusion temperature (T_c_) was conducted using the TRIOS TA-Instrument Thermal Analysis System Software, as outlined in the works of (Andler et al., 2023) and Morales-Quintana et al. (2022b).

### 2.5 Determination of the antioxidant compounds

Total phenolics and flavonoids were quantified in different developmental stages and treated fruits using well-established methodologies. Three independent extractions were performed on 10 g of each sample, representing the different treatments and developmental stages. These extracts were subsequently analyzed for total phenolic and flavonoid content following the procedures outlined by Parra-Palma et al. (2020). Firstly, the total phenolic content was evaluated diluted extracts were oxidized with the Folin–Ciocalteu reagent, neutralized with sodium carbonate, and the absorbance of the blue coloration was measured at 700 nm after 30 minutes using a Multiskan SkyHigh Microplate Spectrophotometer (Thermo Fisher Scientific). A standard curve of gallic acid was used for quantification, and results were expressed as grams of gallic acid equivalents per kilogram of fruit (g kg^-1^ GAE). Data represent the means ± standard errors (SEs) of three biological replicates, each with two technical replicates. Respect to the total flavonoid content, it was determined using the aluminum chloride colorimetric method adapted from Chang et al. (2002), with quercetin as the calibration standard. A 0.5 mL aliquot of each diluted extract was mixed with 1.5 mL of 95% ethanol, 0.1 mL of 10% aluminum chloride, 0.1 mL of 1 M potassium acetate, and 2.8 mL of distilled water. After 30 minutes of incubation at room temperature, absorbance was measured at 415 nm using the same microplate spectrophotometer. Results were expressed as grams of quercetin equivalents per kilogram of fruit (g kg^-1^ QE) and represent the means ± SEs of three biological replicates with two technical replicates. Finally, the total anthocyanin content was quantified using the differential pH method and expressed as cyanidin-3-O-glucoside equivalents per 100 mg of fresh fruit weight (C3GE/100 mg FW), following the methodology described by Parra-Palma et al. (2020).

### 2.6 Total antioxidant capacity of the fruits

The antioxidant capacity of the treated and developmental fruit stage extracts was assessed using the DPPH radical scavenging assay, following the method described by Cheel et al. (2007). Briefly, 20 μL of the extract was mixed with 0.5 mL of 0.5 mM DPPH solution and 0.5 mL of 100 mM acetate buffer (pH 5.5). The reaction mixtures were prepared in triplicate, and absorbance was measured at 517 nm using an Epoch 2 microplate spectrophotometer. Radical scavenging ability was calculated by comparing the samples to a methanol-DPPH negative control. The ferric reducing antioxidant power (FRAP) assay was also employed, based on the methodology of Parra-Palma et al. (2020). In this assay, methanol extracts were reacted with a ferric-tripyridyltriazine (Fe³^+^-TPTZ) complex, which antioxidants reduce to the ferrous form (Fe²^+^-TPTZ), resulting in a blue coloration measured spectrophotometrically, and using Trolox for the calibration curve. FRAP values provided a quantitative measure of reducing power and antioxidant potential.

### 2.7 Antioxidant enzyme activity

Ripe fruits (500 mg fresh weight) were ground in liquid nitrogen, and the resulting powder was homogenized in a pre-chilled 50 mM phosphate buffer (pH 7.8) containing 1% polyvinylpyrrolidone (PVP). The homogenates were centrifuged at 10,000 rpm for 20 minutes at 4°C, and 5 mL of the supernatant was collected for enzymatic assays. Activities of superoxide dismutase (SOD), catalase (CAT), peroxidase (POD), and ascorbate peroxidase (APX) were determined following the protocols previously standardized by Morales-Quintana et al. (2022a). All determinations were conducted in biological triplicates to ensure reliability. Briefly, in the apoplastic fractions obtained from frozen cell samples were measured spectrophotometrically. The CAT activity was measured by monitoring the decrease in absorbance at 240 nm in 50 mM phosphate buffer (pH 7.5) containing 20 mM H_2_O_2_ (Erdogan et al., 2016). APX activity was determined by following the decrease in A290 (extinction coefficient 2.8 mM^-1^ cm^-1^) for 1 min in 1 mL of a reaction mixture containing 50 mM potassium phosphate buffer (pH 7.0), 0.5 mM ascorbic acid, 0.1 mM H_2_O_2_ and 200 µl of enzyme extract (Erdogan et al., 2016). Respect to the POP activity, it was measured by monitoring the increase in absorbance at 470 nm in 50 mM phosphate buffer (pH 5.5) containing 1 mM guaiacol and 0.5 mM H_2_O_2_ (Guo et al., 2010; Erdogan et al., 2016). Finally, The SOD activity was estimated by recording the decrease in optical density of nitro-blue tetrazolium dye by the enzyme (Guo et al., 2010; Erdogan et al., 2016).

### 2.8 Statistical analysis and experimental design

The following experimental design was implemented, utilizing control groups composed of fruits at various stages examined in the research, alongside an experimental group exposed to MeJA at diverse stages of the fruits. The results, acquired in triplicate, were represented as average values ± standard deviation. The statistical analysis was conducted utilizing statistical software by SPSS Inc. (Chicago, IL, version 15). An ANOVA test was employed to compare the average values among groups, with a significance threshold established at p < 0.05.

## 3 Results

### 3.1 Fruits physiological evaluations

Firstly, a comprehensive analysis of blueberry (*V. corymbosum*) fruit characteristics reveals significant changes in color, weight, and diameter across the ripening stages (green, pink, blue) over two agronomic seasons (**Table 1**). The CIELAB color parameters (L*, a*, b*) indicate a progressive shift in fruit color as ripening advances. Specifically, the L value* (lightness) decreases from the green to blue stage, suggesting that the fruit becomes darker as chlorophyll degrades and anthocyanins accumulate in the two seasons (**Table 1**). The a value* transitions from negative (indicating green) in the green stage to positive in the pink and blue stages, highlighting the increase in red pigmentation associated with anthocyanin synthesis (**Table 1**). Meanwhile, the b value* decreases, reflecting a reduction in yellow hues and a shift towards a blue-purplish tone, which is characteristic of mature blueberries (**Table 1**). The calculated ΔE* values, which indicate color change from the green stage, are highest between green and blue stages, confirming significant color transitions during ripening (**Table 1**).

**Table 1:** Physiological parameter of the different fruit ripening stage of blueberry (*Vaccinium corymbosum*) evaluation during two agronomical seasons.

Season	Stage	CIELAB color space			ΔE*	Weight (g)	Diameter (mm)
		L*	a*	b*			
2022	Green	25.99 ± 9.53	-2.66 ± 4.53	23.47 ± 7.55	–	1.23 ± 0.20 ^b^	14.28 ± 0.83 ^b^
	Pink	12.81 ± 7.39	10.87 ± 8.92	6.64 ± 6.21	23.97	1.38 ± 0.30 ^b^	14.28 ± 1.16 ^b^
	Blue	18.55 ± 10.33	-3.13 ± 10.31	0.35 ± 5.17	24.26	1.99 ± 0.32 ^a^	16.57 ± 1.17 ^a^
2023	Green	11.52 ± 6.42	-3.03 ± 2.69	11.12 ± 7.77	–	0.64 ± 0.17 ^b^	11.20 ± 1.10 ^b^
	Pink	18.23 ± 9.92	10.13 ± 9.00	11.09 ± 10.21	14.77	1.48 ± 0.28 ^b^	15.23 ± 1.43 ^b^
	Blue	13.20 ± 7.09	7.67 ± 8.19	1.34 ± 4.73	14.59	1.73 ± 0.32 ^a^	16.10 ± 1.27 ^a^

Values indicate the mean of thirty replicates, and the standard deviations are also shown. Different superscript alphabets in a column represent a statistically significant difference between the means (p < 0.05).

*Values compared to the green stage in each season.

Respect to the size of the fruit during the developmental stages, in 2022, fruits at the blue stage exhibit significantly higher weight and diameter (1.99 g and 16.57 mm) compared to the green (1.23 g and 14.28 mm) and pink (1.38 g and 14.28 mm) stages (**Table 1**). A similar trend is observed in 2023, with blue-stage fruits reaching 1.73 g in weight and 16.10 mm in diameter, confirming the physical enlargement and mass increase that typically accompany blueberry ripening (**Table 1**). The green-stage fruits in 2023 had lower average weight (0.64 g) and diameter (11.20 mm) compared to 2022, possibly reflecting environmental or agronomic factors affecting fruit development this season (**Table 1**). This form, the fruit transitions from green to blue-purple stage, reflecting chlorophyll degradation and pigment synthesis, while increasing in size and weight due to cell expansion. Simultaneously, the breakdown of hemicellulose and pectin leads to softening, enhancing fruit texture. Seasonal variations in these traits highlight the influence of environmental factors, underscoring the importance of optimizing agronomic practices to ensure consistent fruit quality and yield.

**Figure 2:**
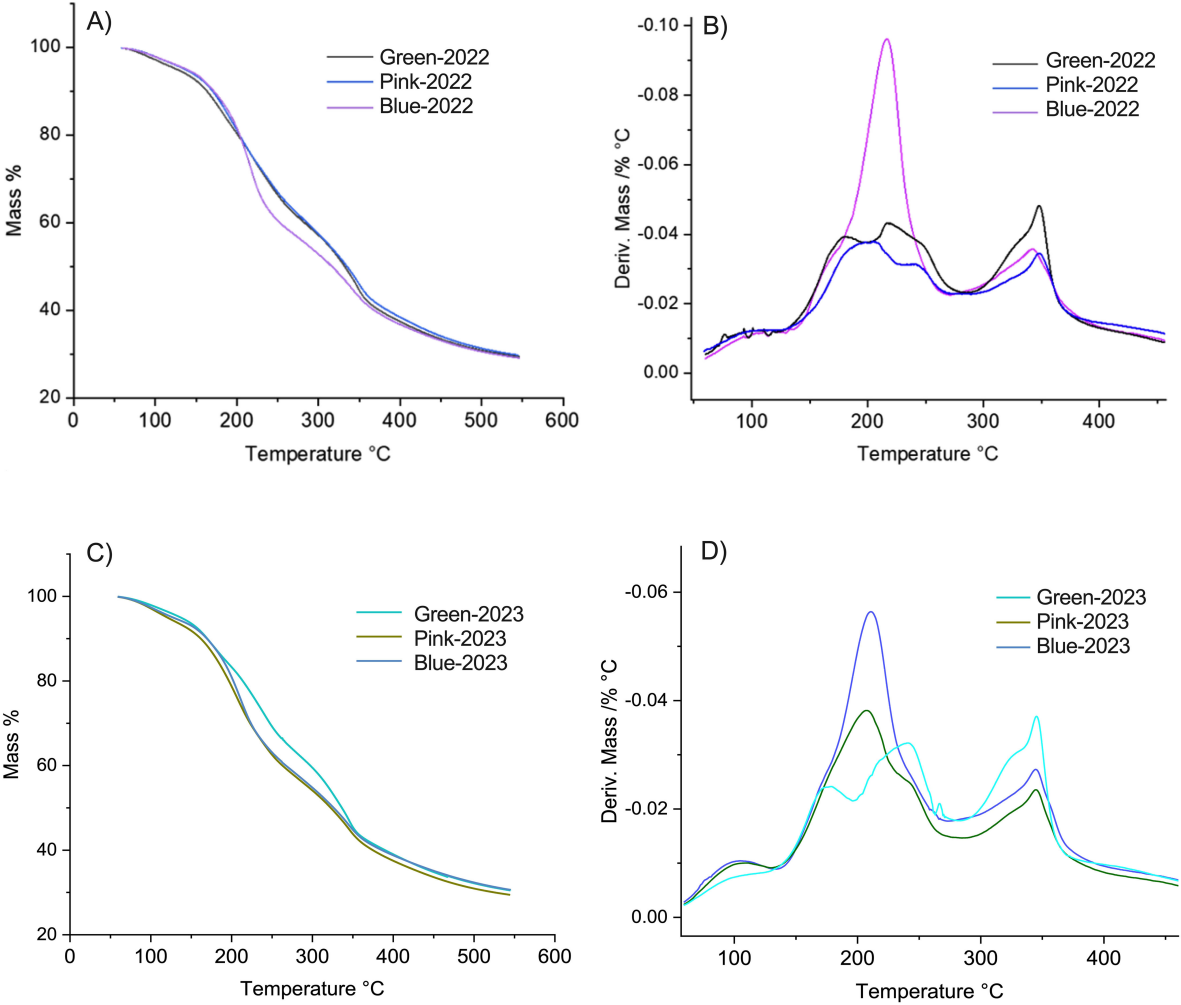
Thermogravimetric analysis (TGA) and derivative thermogravimetry (DTG) curves of blueberries at different developmental stages from the 2022 and 2023 seasons. **(A)** TGA thermograms representing mass loss (%) as a function of temperature (50–550°C) for blueberries harvested in 2022 at three different developmental stages (Green, Pink, and Blue). The thermal degradation profiles exhibit a continuous weight loss, indicative of different decomposition phases related to moisture evaporation, thermal degradation of organic components, and residual char formation. **(B)** DTG curves derived from **(A)**, showing the rate of mass loss as a function of temperature. The peaks indicate the maximum degradation rates, highlighting key thermal degradation events for each developmental stage in the 2022 season. A distinct peak is observed around 200°C, suggesting variations in the thermal stability of the fruit depending on its ripeness. **(C)** TGA thermograms for blueberries from the 2023 season, following the same developmental stages (Green, Pink, and Blue). The mass loss patterns are similar to those of 2022, with minor differences likely attributed to seasonal variations in fruit composition. **(D)** DTG curves corresponding to **(C)**, illustrating the temperature points at which the highest degradation rates occur. Compared to 2022, the degradation peaks in 2023 exhibit slight shifts, which may be associated with differences in biochemical composition, water content, or structural changes during fruit maturation.

Additionally, the cell wall stability was determined using a thermogravimetric analysis (TGA) and derivative thermogravimetry (DTG), according to Castro and Morales-Quintana (2019). The TGA and DTG results in **Figure 2** show how different cell wall components in the samples degrade at specific temperature ranges, which can give us insights into how the structure of the cell wall changes and relates to fruit softening according to the development stage. As the temperature increases, we observe different stages of weight loss, which correspond to the breakdown of various parts of the cell wall, including water loss and the degradation of important polymers like cellulose and hemicellulose (**Figure 2**). These polymers provide rigidity and structure, so when they break down, the cell wall becomes softer (Castro and Morales-Quintana, 2019). As shown in **Figure 2**, in the temperature range of 200 to 330°C, the green stage exhibited greater firmness compared to the ripe (blue) stage. Notably, differences were observed in the behavior of the pink stage, where in the 2022 season, its thermal profile resembled that of the green stage, while in the 2023 season, it was more like the blue stage. This suggests that the blue stage is more depolymerized than the green stage, which demonstrated higher thermal stability of the cell wall (**Figures 2A, C**). According to the data described by Castro and Morales-Quintana (2019), the region corresponds to the decomposition of hemicellulose fractions between 200 and 300°C (Xiao et al., 2001) and shorter-chain pectin fractions at approximately 250°C (Ghaffari et al., 2007) (**Figures 2B, D**).

### 3.2 Flavor, antioxidant, and enzymatic dynamics in blueberry across developmental stages

In terms of SSC (an indicator of sugar content described in °Brix), the blue stage shows the highest values, especially in 2022 where it measured 18.5 ± 0.1°Brix, aligning with the increased sweetness typically associated with fruit ripening (**Supplementary Figure S1**). TA decreases across the developmental stages, with 1.0 ± 0.015%TA in green stage of 2022 season to 1.7 ± 0.011%TA in blue stage of 2022, reflecting a reduction in acidity as the fruit ripe (**Supplementary Figure S1**). Consequently, the SSC/TA ratio—a critical measure of fruit taste quality—rises sharply, especially in the pink and blue stages (**Supplementary Figure S1**). This ratio increase suggests a shift from acidic to sweeter flavors, which are more desirable in ripe fruits.

**Figure 3:**
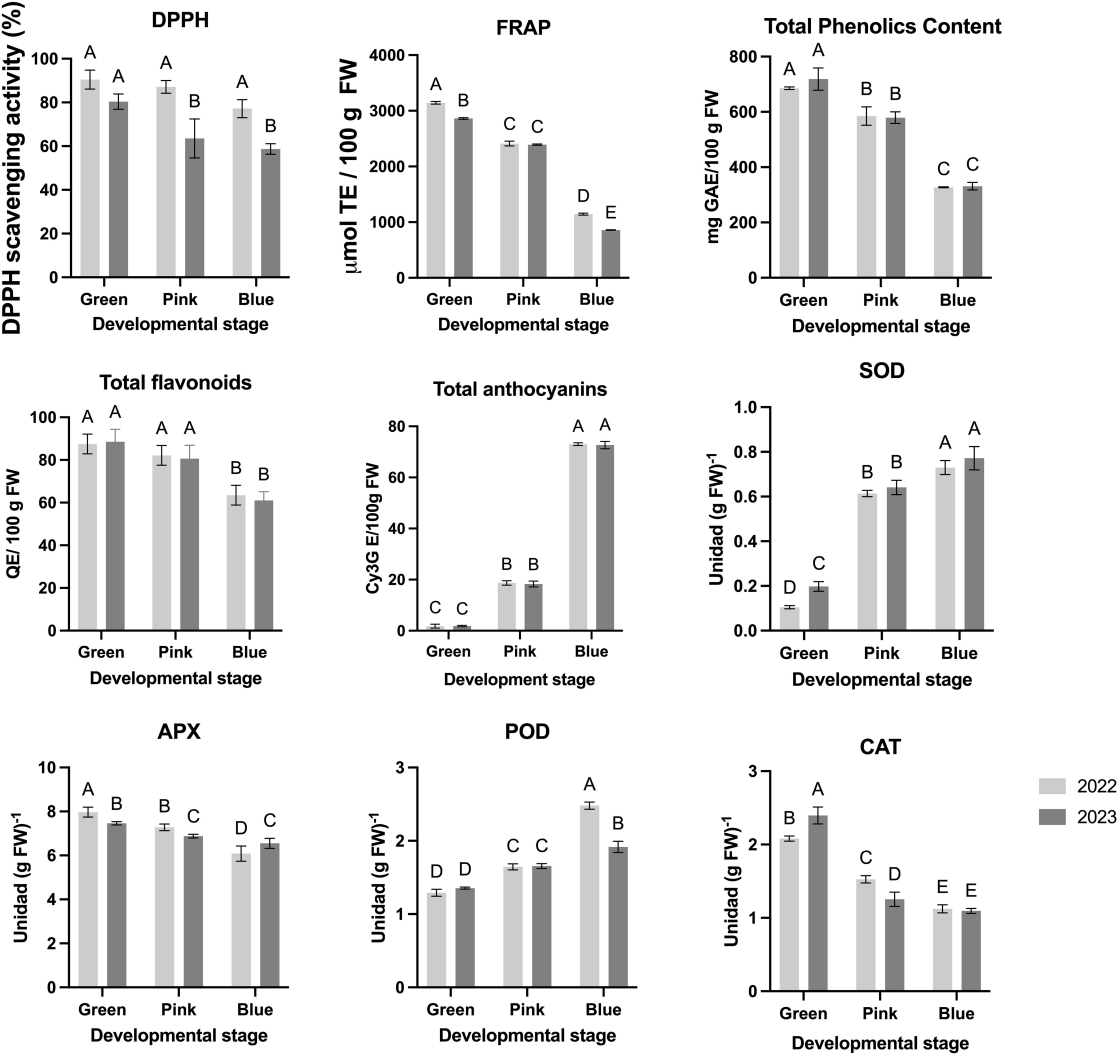
Nutritional and antioxidant parameters of the blueberry fruits during three different developmental stage and two different seasons. Different letters indicate significant differences (P < 0.05; two-way ANOVA). Bars represent means SE from three independent experiments.

The antioxidant capacity of blueberry fruits at different developmental stages (green, pink, and blue) was evaluated through DPPH radical scavenging activity and ferric reducing antioxidant power (FRAP) assays over two consecutive years (2022 and 2023) (**Figure 3**). The DPPH scavenging activity indicates a strong antioxidant potential at the green stage, with values near 100% in both years (**Figure 3**). This activity significantly declines as the fruit matures, particularly at the blue stage, where a notable decrease is observed in 2023 compared to 2022. These findings suggest that early-stage blueberries possess high radical-scavenging capacity, which diminishes as the fruit ripens, potentially due to the degradation or transformation of phenolic compounds (**Figure 3**). Similarly, the FRAP results reveal a high antioxidant potential in the green stage, reaching values close to 3,000 µmol TE/100 g FW. As with DPPH, FRAP values decrease progressively through the pink and blue stages, with the lowest values recorded in the blue stage for both years (**Figure 3**). The significant decline in FRAP at later stages aligns with the ripening process, which is associated with reduced levels of antioxidant compounds as total phenols (**Figure 3**).

The total phenolic content demonstrates a clear decline as the fruit progresses from the green to the blue stage, with the highest concentration observed at the green stage for both years (**Figure 3**). Flavonoid Content remains relatively stable across stages in both years, with minor differences observed between developmental stages, indicating that flavonoid accumulation is less affected by ripening compared to phenolics (**Figure 3**). Respect to the total Anthocyanins exhibit a marked increase by the blue stage, reflecting anthocyanin accumulation (**Figure 3**) linked to the color change associated with ripening (**Table 1**). Anthocyanins are significantly more concentrated in the blue stage than in earlier stages, underscoring their role in late-stage pigmentation.

The enzymatic activity of antioxidant enzymes—ascorbate peroxidase (APX), catalase (CAT), peroxidase (POD), and superoxide dismutase (SOD)—was assessed across developmental stages (green, pink, and blue) in blueberry fruits for two consecutive years (2022 and 2023), highlighting the fruit’s dynamic response to oxidative stress during ripening (**Figure 3**). APX activity is notably higher at the green stage, reaching approximately 8 units/g FW in both years, but statistically decreases progressively through the pink and blue stages (**Figure 3**). This trend suggests an early peak in APX activity as the fruit initiates protective mechanisms against oxidative stress during the initial stages of maturation. Similarly, CAT activity is highest at the green stage in both years (over 2 units/g FW), then declines significantly as the fruit transitions to the pink and blue stages, indicating a reduction in CAT-mediated hydrogen peroxide scavenging as ripening advances (**Figure 3**). In contrast, POD activity shows a peak at the blue stage, with a significant increase from the green and pink stages, especially in 2022. This late rise in POD activity suggests an adaptive response that may play a role in the final stages of fruit maturation and protection against oxidative degradation during full ripeness (**Figure 3**). SOD activity, essential for dismutation of superoxide radicals, increases as the fruit matures, reaching similarly high levels at the pink and blue stages in both years. The progressive rise in SOD aligns with the increased metabolic demands and potential oxidative stress encountered as the fruit reaches maturity (**Figure 3**).

### 3.3 Different number of the MeJA applications in the blueberry plants and the effect over the fruits obtained

**Figure 4:**
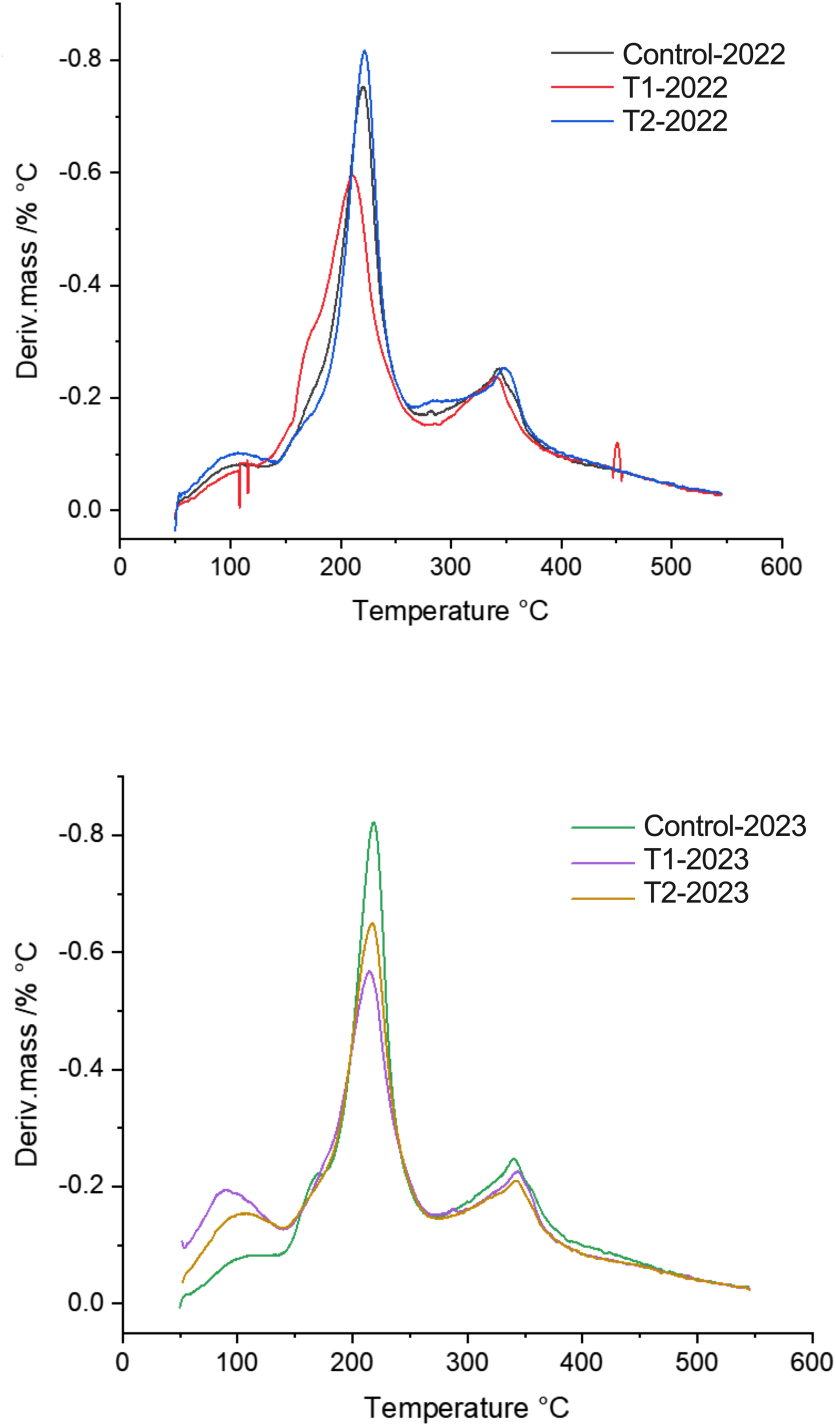
First derivative of the thermogram curves (TG/DTG thermogram) showing the maximum degradation temperatures from two different MeJA treatment (T1 and T2), during two season 2022 and 2023. DTG curves showing the rate of mass loss as a function of temperature. The peaks indicate the maximum degradation rates, highlighting key thermal degradation events for each developmental stage in the 2022 and 2023 seasons. A distinct peak is observed around 200°C, suggesting variations in the thermal stability of the fruit depending on its ripeness.

MeJA applications influenced multiple physiological, biochemical, and enzymatic parameters in blueberry fruits across two consecutive seasons (2022 and 2023). While thermogravimetric analysis showed no significant differences between treatments, minor variations around 200°C (**Figure 4**), suggested changes in hemicellulose structure, according to Xiao et al. (2001) and validated by our laboratory in Castro and Morales-Quintana (2019).

**Figure 5:**
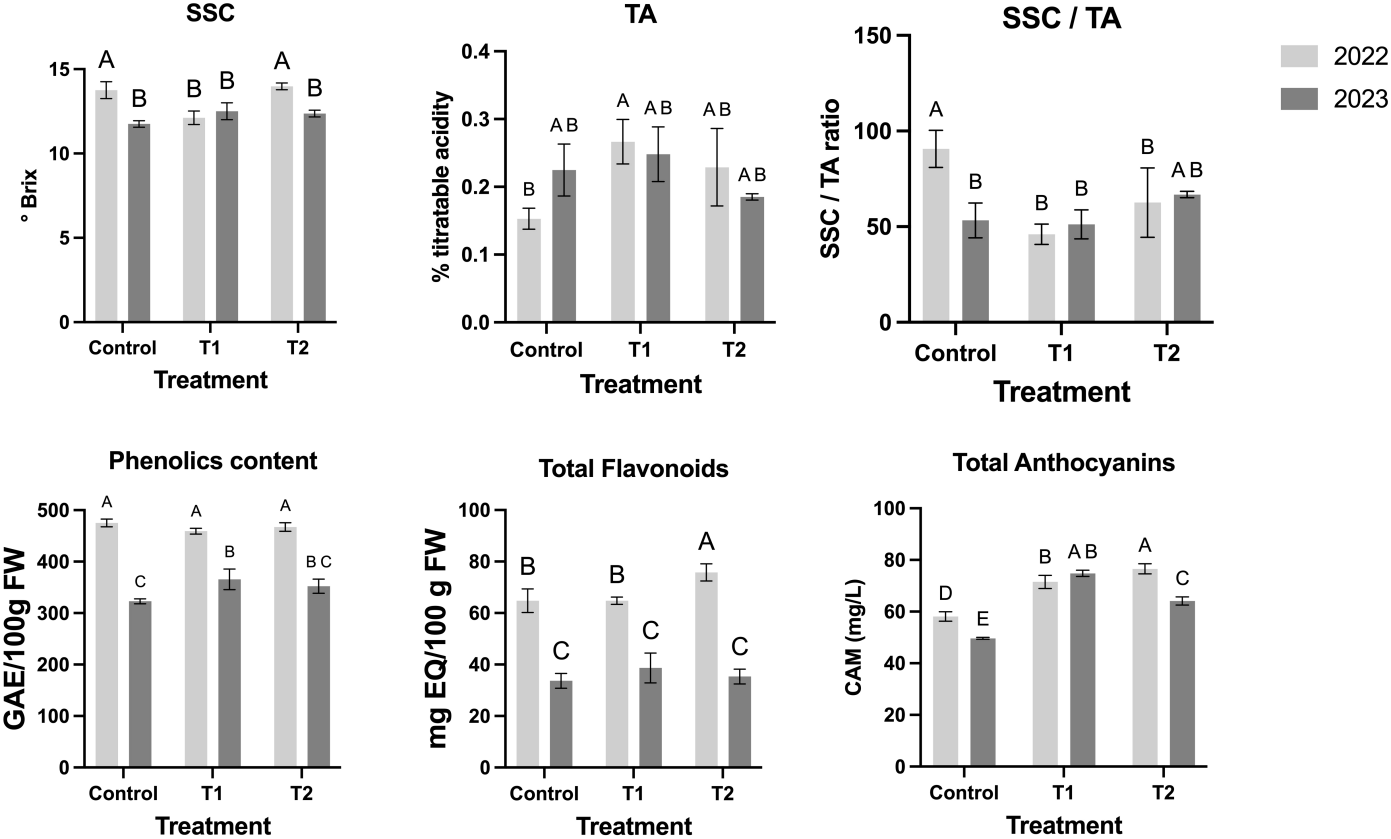
MeJA treatment over blueberry plants and the effect over the nutritional and physiological fruits parameters. Different letters indicate significant differences (P < 0.05; two-way ANOVA). Bars represent means SE from three independent experiments.

Single MeJA (T1) led to a reduction in SSC in 2022, suggesting lower sugar accumulation (**Figure 5**). TA varied across years, increasing significantly in T1 during 2022 but showing a slight decline in T2 in 2023 (**Figure 5**). Consequently, the SSC/TA ratio, a key indicator of fruit sweetness, was lower in treated fruits, particularly in 2022 (**Figure 5**). For the color parameters in 2022, the control group displayed L*, a*, and b* values of 5.88 ± 3.40, 1.25 ± 2.36, and -0.55 ± 1.51, respectively. Treatment T1 showed increased L* and altered color attributes, with values of 15.98 ± 11.50 for L*, -0.46 ± 9.23 for a*, and -0.72 ± 5.55 for b*, corresponding to a color difference (ΔE*) of 10.24. In T2, these values were 7.48 ± 6.34, -0.40 ± 3.94, and -0.08 ± 2.31, with ΔE* at 2.35 (**Table 2**). The average fruit weight and diameter remained statistically consistent across treatments, with slight variances (e.g., 1.46 ± 0.39 g and 14.62 ± 1.42 mm in the control) (**Table 2**). In 2023, the control group values were 12.63 ± 3.55 for L*, 3.88 ± 5.44 for a*, and 0.62 ± 3.32 for b*. T1 had color parameters of 18.14 ± 5.22 (L*), 3.54 ± 5.93 (a*), and -1.76 ± 2.82 (b*), with ΔE* of 6.01, while T2 displayed L*, a*, and b* values of 12.70 ± 4.48, 1.83 ± 3.21, and 0.43 ± 2.24, with ΔE* of 2.06 (**Table 2**). Across both seasons, treatment effects on weight and diameter showed non-significant differences compared to the control group, with values in T1 and T2 generally within the range of 1.46–2.16 g and 14.62–17.40 mm, respectively (**Table 2**).

**Table 2:** Physiological parameters of blueberry (*Vaccinium corymbosum*) fruits obtained from plants after different treatments with MeJA, during 2 agronomic harvests seasons.

Season	Stage	CIELAB color space			ΔE*	Weight (g)	Diameter (mm)
		L^*^	a^*^	b^*^			
2022	Control	5.88 ± 3.40	1.25 ± 2.36	-0.55 ± 1.51	–	1.46 ± 0.39 ^a^	14.62 ± 1.42 ^a^
	T1	15.98 ± 11.50	-0.46 ± 9.23	-0.72 ± 5.55	10.24	1.66 ± 0.35 ^a^	15.65 ± 1.31 ^a^
	T2	7.48 ± 6.34	-0.40 ± 3.94	-0.08 ± 2.31	2.35	1.46 ± 0.35 ^a^	16.65 ± 11.28 ^a^
2023	Control	12.63 ± 3.55	3.88 ± 5.44	0.62 ± 3.32	–	1.75 ± 0.24 ^a^	16.00 ± 0.98 ^a^
	T1	18.14 ± 5.22	3.54 ± 5.93	-1.76 ± 2.82	6.01	2.16 ± 0.33 ^a^	17.40 ± 1.25 ^a^
	T2	12.70 ± 4.48	1.83 ± 3.21	0.43 ± 2.24	2.06	1.93 ± 0.21 ^a^	16.53 ± 0.97 ^a^

Values indicate the mean of thirty replicates, and the standard deviations are also shown.

*In ΔE, the values compared to the control samples in each season.

In the weight and diameter column, the superscript ‘a’ indicates that there are no significant differences with a p<=0.95.

In terms of phenolic composition, total phenolic content remained stable across treatments in 2022 but increased in T1 during 2023 (**Figure 5**). Flavonoid content was higher in T2 in 2022, whereas no differences were observed in 2023 (**Figure 5**). Anthocyanin accumulation increased with MeJA applications, particularly in T2, suggesting enhanced pigmentation and antioxidant potential (**Figure 5**).

**Figure 6:**
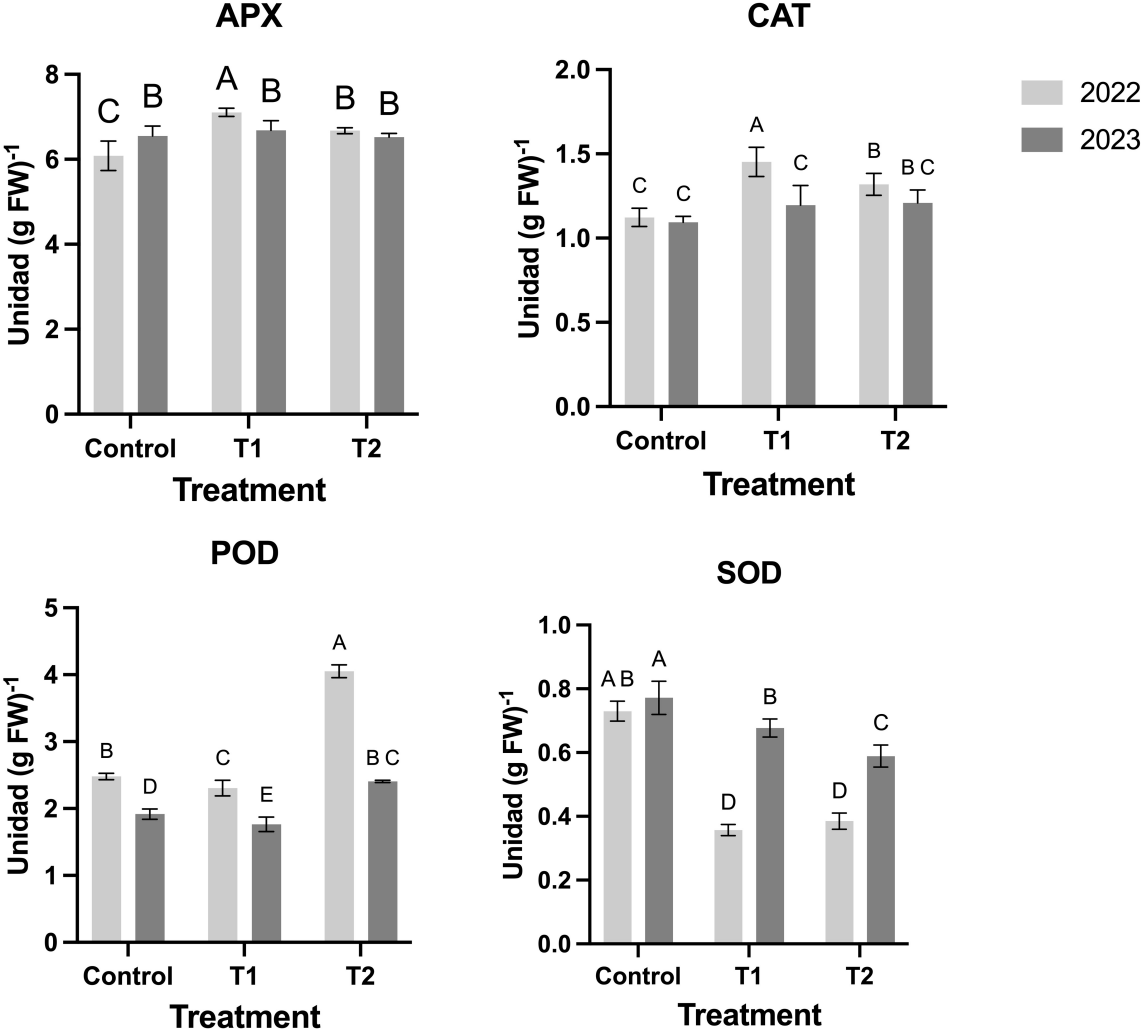
Effect of MeJA treatment on the activities of the antioxidant enzymes (CAT, POD, SOD, and APX) in the blueberry fruits. Different letters indicate significant differences (P < 0.05; two-way ANOVA). Bars represent means ± SE from three independent experiments.

MeJA treatments also influenced antioxidant enzyme activities. Ascorbate peroxidase (APX) and catalase (CAT) increased significantly in T1, particularly in 2022, while T2 exhibited a slight decline, indicating a possible saturation effect with repeated applications (**Figure 6**). Peroxidase (POD) responded more strongly to T2, suggesting a role in cell wall reinforcement (**Figure 6**). Superoxide dismutase (SOD) was higher in T1 than in T2, implying that a single MeJA application may be sufficient to trigger an optimal antioxidative response (**Figure 6**).

**Figure 7:**
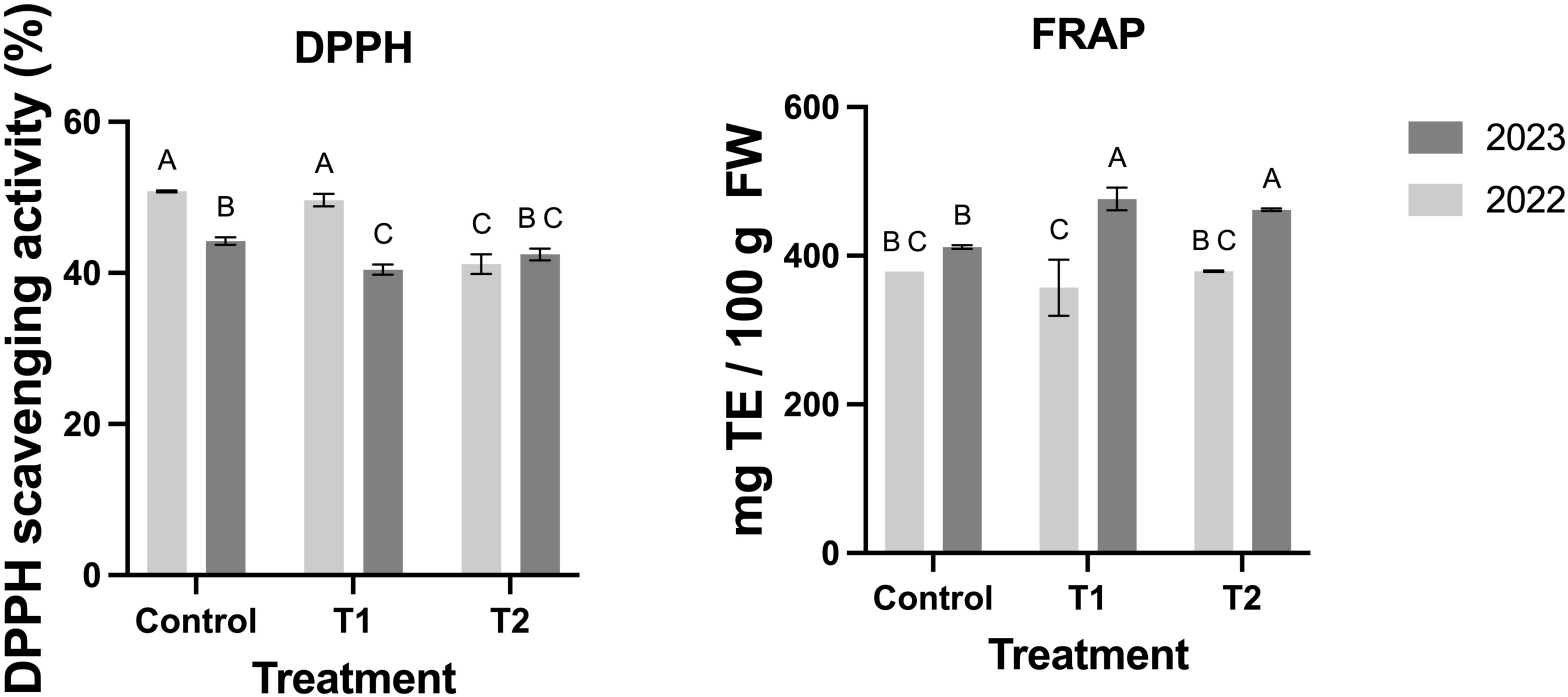
Effect of MeJA treatment on the activities of the antioxidant capacity in the blueberry fruits. Different letters indicate significant differences (P < 0.05; two-way ANOVA). Bars represent means ± SE from three independent experiments.

Additionally, antioxidant capacity, measured through DPPH and FRAP assays, improved with MeJA treatments (**Figure 7**). While FRAP values were higher in T1 and T2 during 2023, no significant differences were detected in 2022 (**Figure 7**). DPPH scavenging activity showed a progressive increase with MeJA, particularly in T2, indicating enhanced free radical neutralization (**Figure 7**).

Overall, MeJA treatments modified fruit physiology, biochemical composition, and antioxidant activity, with T1 generally inducing stronger metabolic changes, while T2 resulted in more pronounced antioxidant responses, particularly in anthocyanin accumulation and peroxidase activity.

Finally, a Pearson’s correlation analysis reveals key relationships among the measured parameters in MeJA-treated blueberry fruits across the two seasons. In the 2022 season, phenolics content showed strong negative correlations with CAT (-0.99) and APX (-1.00) activities, suggesting that higher phenolic levels may reduce the need for these antioxidant enzymes (**Table 3**). Total flavonoids were strongly positively correlated with POD activity (1.00) but negatively with DPPH (-0.99), indicating a potential trade-off between flavonoid accumulation and DPPH scavenging capacity (**Table 3**). In the 2023 season, phenolics content was strongly positively correlated with total anthocyanins (0.99), SSC (0.99), and FRAP (1.00), highlighting the role of phenolics in enhancing sugar content, anthocyanin accumulation, and overall antioxidant capacity (**Table 3**). These results underscore the complex interplay between phenolic compounds, antioxidant enzymes, and fruit quality parameters in response to MeJA treatment.

**Table 3:** Pearson’s correlation analysis of phenolics content, total flavonoids, total anthocyanins, SSC, TA, SSC/TA, CAT, APX, POD, SOD, DPPH, and FRAP in four different MeJA treatment fruits samples.

2022	Phenolics content	Total Flavonoids	Total Anthocyanins	SSC	TA	SST/TA	CAT	APX	POD	SOD	DPPH	FRAP
Phenolics content												
Total Flavonoids	0,00											
Total Anthocyanins	-0,70	0,71										
SSC	0,80	0,59	-0,14									
TA	-0,98	0,19	0,83	-0,67								
SSC/TA	0,99 *	-0,15	-0,80	0,71	-1,00 *							
CAT	-0,99 *	0,11	0,78	-0,73	1,00 *	-1,00 *						
APX	-1,00 *	0,09	0,76	-0,75	0,99 *	-1,00 *	1,00 *					
POD	0,09	1,00 *	0,65	0,67	0,10	-0,06	0,02	0,00				
SOD	0,90	-0,44	-0,94	0,46	-0,97 *	0,95	-0,94	-0,93	-0,36			
DPPH	0,11	-0,99 *	-0,79	-0,50	-0,30	0,26	-0,22	-0,20	-0,98 *	0,54		
FRAP	0,86	0,52	-0,23	1,00 *	-0,74	0,77	-0,79	-0,81	0,59	0,54	-0,42	
2023	Phenolics content	Total Flavonoids	Total Anthocyanins	SSC	TA	SST/TA	CAT	APX	POD	SOD	DPPH	FRAP
Phenolics content												
Total Flavonoids	0,92											
Total Anthocyanins	0,99 *	0,96										
SSC	0,99 *	0,85	0,96									
TA	0,16	0,54	0,28	0,01								
SSC/TA	0,09	-0,31	-0,04	0,24	-0,97 *							
CAT	0,91	0,67	0,85	0,96	-0,27	0,50						
APX	0,62	0,88	0,71	0,50	0,88	-0,73	0,23					
POD	-0,02	-0,41	-0,14	0,13	-0,99 *	0,99 *	0,40	-0,80				
SOD	-0,69	-0,35	-0,59	-0,79	0,61	-0,78	-0,93	0,15	-0,71			
DPPH	-0,97	-0,99 *	-0,99 *	-0,92	-0,40	0,16	-0,78	-0,79	0,27	0,48		
FRAP	1,00 *	0,88	0,98	1,00 *	0,07	0,18	0,94	0,54	0,08	-0,75	-0,94	

*indicate significant at 0.01 p<value Fisher’s least significant difference (LSD) test.

## 4 Discussion

The results of this study reveal the complex biochemical, physiological, and structural changes occurring in blueberry (*V. corymbosum*) fruits during ripening and following MeJA treatments. These findings significantly enhance our understanding of how hormonal treatments influence fruit quality, nutritional value, and cell wall integrity, offering valuable insights for both production and postharvest management during two productive seasons. In this line, when evaluating the effects of MeJA on blueberries over the 2022 and 2023 growing seasons, it’s essential to consider environmental factors that may have influenced the observed differences in responses. Variations in climate conditions between these seasons can significantly impact plant physiology and the efficacy of treatments like MeJA, when these are carried out in open productive commercial orchard exposed to the environment. For instance, temperature fluctuations can affect the rate of plant metabolic processes, potentially altering the plant’s response to MeJA. Differences in rainfall, with 480.2 mm of rainfall during 2022 and 678.4 mm of rainfall during 2023 in Cauquenes (Information available at https://agrometeorologia.cl/), its can influence disease prevalence and stress levels in plants, particularity during 2022 season which may interact with MeJA treatment outcomes. Additionally, the number of chilling hours (periods of cold temperatures) varies annually and is vital for blueberry bud development; insufficient chilling can lead to poor bud break and uneven ripening, which may alter the plant’s responsiveness to MeJA treatments.

Now, respect to the thermogravimetric analysis (TGA), it’s have been widely used in different investigations, including a study of the changes in the physiological properties of the cell wall during ripening of the commercial strawberry fruit, and showed a lower thermal stability at the ripe stage than at the green stage (Castro and Morales-Quintana, 2019). Additionally, a second work was published, when we evaluated the changes in cell wall thermal stability produced by the abscisic acid (ABA) hormone and showed that ABA treatment produced a lower thermal stability in strawberry fruit than the control treatment (Castro et al., 2021a). Now, we showed the thermal analysis (**Figure 2**), that provides further insights into structural changes in the cell wall during ripening in blueberries fruits. As the cell wall undergoes gradual degradation, the fruit softens, losing firmness. The degradation patterns observed through temperature-based analysis highlight which components of the cell wall confer stability over time, offering a deeper understanding of the natural softening process in ripening fruits. This technique proves valuable for studying both thermal stability and the structural transformations associated with fruit texture, as well as providing evidence of the effect of MeJA on increasing the stability of the different polymers in the cell wall, which would serve as a starting point for further studies on post-processing and drying of fruits.

**Table 1** highlights the intricate relationships between color, size, and developmental stages in blueberries, with each ripening stage exhibiting distinct physiological profiles that vary between seasons. MeJA application has been shown to promote anthocyanin biosynthesis (**Figure 3**), leading to intensified blue pigmentation in ripe fruits. In red-blushed pears, MeJA treatment increased anthocyanin content in the peels, resulting in enhanced coloration (Li et al., 2023). This effect is attributed to the upregulation of genes involved in the anthocyanin biosynthetic pathway, as evidenced by increased expression of both structural and regulatory genes following MeJA application (Li et al., 2023), while in terms of acceptability, it could generate greater consumer appeal.

Variations in phenolic content observed in the study respect to the different seasons, could be attributed to environmental factors, particularly sunlight exposure, which is a well-documented regulator of phenolic biosynthesis in plants. Sunlight, especially UV-B radiation, stimulates the phenylpropanoid pathway, leading to increased accumulation of flavonoids and other phenolic compounds as part of the plant’s protective mechanisms against oxidative stress (Zoratti et al., 2014). In this study, fruits exposed to direct sunlight exhibited a 23.5% increase in total phenolic content compared to those grown in shaded conditions. This trend aligns with findings in blueberries, where in the control samples exist differences between two seasons, and according to the According to data obtained from the INIA experimental station, the average solar radiation value during 2022 was higher than in 2023, with values ​​of 16.1 Mj/m² and 15.5 Mj/m², respectively (Information available at https://agrometeorologia.cl/).

These data emphasize the importance of monitoring such parameters for quality control in blueberry production, as they directly affect consumer preference and marketability. As the fruits progress from the green to the blue ripening stage, phenolic content and antioxidant capacity (DPPH and FRAP assays) decline, while anthocyanin content increases (**Figure 3**). This trend aligns with prior studies showing that ripening reduces antioxidant metabolites such as phenolics, whereas anthocyanins accumulate to enhance pigmentation. These findings underscore the metabolic trade-offs during maturation, where antioxidant defenses are balanced with physiological requirements such as fruit coloration and sweetness development. Early-stage fruits (green stage) demonstrate the highest radical scavenging activity, making them particularly advantageous for applications prioritizing health-promoting properties over sensory attributes. The increase in DPPH and FRAP activity observed in MeJA-treated fruits is likely associated with the activation of the phenylpropanoid pathway, which leads to the accumulation of bioactive compounds with strong radical-scavenging properties (Delgado et al., 2018; Zuñiga et al., 2020). MeJA has been shown to induce key enzymes such as phenylalanine ammonia-lyase (PAL), chalcone synthase (CHS), and peroxidase (POD), all of which contribute to enhanced antioxidant responses (Wasternack, 2007). Moreover, the consistently higher antioxidant activity in MeJA-treated fruits aligns with findings from similar studies in other crops. For instance, strawberries (*Fragaria × ananassa*) treated with MeJA exhibited increased total phenolic and flavonoid content, leading to improved antioxidant activity and delayed senescence (Zuñiga et al., 2020).

Biochemically, MeJA applications influenced key nutritional traits (**Figure 5**). While total phenolic and flavonoid contents showed year-dependent variability, T2 consistently enhanced anthocyanin accumulation, which correlated with the blue stage’s pigmentation. These results align with the role of MeJA in activating the biosynthesis of secondary metabolites such as anthocyanins, which contribute to both fruit coloration and antioxidant properties. Additionally, MeJA-treated fruits displayed lower SSC/TA ratios compared to controls, indicating a subtle modulation of sugar and acid metabolism that could impact fruit flavor (**Figure 5**). Additionally, previously MeJA treatment has been shown to enhance the antioxidant capacity of blueberries, which has practical implications for extending their shelf life (Wang et al., 2019). Studies indicate that MeJA application increases the content of non-enzymatic antioxidants, such as phenolics and flavonoids, in blueberries (Wang et al., 2019). This is partially like what was observed for the O’neail cultivar that we have worked on in this study, because the results are like those described by Wang et al. (2019) but only in the case of phenols for the 2022 season, while flavonoids only in the 2023 season (**Figure 5**). This enhancement in antioxidant compounds helps in maintaining fruit quality during storage.

MeJA treatments significantly influenced enzymatic and antioxidant dynamics in blueberry fruits. Single (T1) and double (T2) applications enhanced the activity of antioxidant enzymes (APX, CAT, POD, and SOD), with T2 eliciting the strongest POD response. This aligns with MeJA’s known role in modulating plant defense pathways and improving oxidative stress tolerance. Understanding oxidative stress as the product of the imbalance between the production of ROS and the plant’s ability to detoxify naturally. Antioxidant capacities measured by DPPH and FRAP assays increased substantially with MeJA applications, particularly in T2, indicating that exogenous MeJA stimulates secondary metabolite biosynthesis. These results suggest that MeJA treatments can improve fruit resilience and nutritional quality, further supporting their use in horticultural practices. Specifically, the enzymatic response patterns suggest that single applications (T1) maximize APX, CAT, and SOD activities, while repeated applications (T2) amplify POD activity, contributing to improved oxidative stress tolerance and overall fruit quality during ripening (**Figure 6**). MeJA modulates antioxidant enzymes through the activation of the jasmonic acid signaling pathway (Fernández-Calvo et al., 2011). Previously, various authors demonstrated that exogenous MeJA treatment during postharvest of different fruits can increase the fruit antioxidant capacity. This result was mainly observed by enhanced SOD, CAT, APX, and polyphenol oxidase (PPO) with cinnamyl-alcohol dehydrogenase (CAD) activities (Asghari and Hasanlooe, 2015, 2016; Li et al., 2017; Mustafa et al., 2018; Wang et al., 2019). Now we evaluate three of this five enzymes during fruit development to evaluate the effect over postharvest conditions.

The MeJA perception process begging by cell receptors, which activates transcription factors such as MYC2, that bind to the promoters of genes encoding these antioxidant enzymes, increasing their expression. MeJA promotes the accumulation of ROS, which, at controlled levels, act as signals to further activate the antioxidant response, enhancing the activity of these enzymes. The interaction of MeJA with other hormones such as ABA also strengthens the antioxidant response, particularly under abiotic stress conditions like drought. In this way, the increase in antioxidant enzyme activity helps mitigate cellular damage induced by oxidative stress, contributing to plant tolerance under various adverse conditions.

Elevated anthocyanin levels (**Figure 3**), in blueberries ripe fruits are associated with numerous health benefits due to their potent antioxidant properties. Anthocyanins have been shown to possess antidiabetic, anticancer, anti-inflammatory, antimicrobial, and anti-obesity effects, as well as prevention of cardiovascular diseases (Khoo et al., 2017).

Finally, **Figure 7** demonstrates that the enhanced enzymatic activity and accumulation of antioxidant compounds induced by MeJA treatments translate into significant improvements in the antioxidant capacity of blueberry fruits. The observed interannual variability highlights the influence of environmental factors on fruit responses. Overall, these findings support the use of MeJA as a practical tool for enhancing the nutritional and functional quality of blueberries by modulating their antioxidant profiles and improving their resilience and marketability.

The evidence findings during the two seasons suggest that the enhanced enzymatic activity (**Figure 6**) and accumulation of antioxidant compounds induced by MeJA treatments (**Figure 5**) translate into significant improvements in the antioxidant capacity of blueberry fruits (**Figure 7**). Variations between the two years may reflect environmental influences on fruit responses. Overall, this evidence supports the use of MeJA as a practical tool to improve the nutritional and functional quality of blueberries by modulating their antioxidant profiles.

While MeJA and ABA have been studied for their roles in fruit ripening and quality enhancement, their effects can vary. For instance, MeJA has been shown to induce resistance to postharvest pathogens, whereas ABA does not significantly affect pathogen development during storage. Auxins, on the other hand, may delay ripening, which could be advantageous or detrimental depending on the desired outcome (Wang et al., 2018, 2020). In this line, recently we have recently published the effect of ABA and MeJA applications on ‘legacy’ blueberry plants and their ability to tolerate water deficit (Balbontín et al., 2025). However, these approaches have been carried out in greenhouse plants, like the large number of the extensive bibliography that can be found on ABA, MeJA and/or Auxin applications. For this reason, our work provides some new approaches to how MeJA can positively affect fruit development and its organoleptic properties.

## 5 Conclusions

This study provides a comprehensive evaluation of the biochemical, physiological, and structural changes occurring in blueberry (*V. corymbosum*) fruits during ripening and following methyl jasmonate (MeJA) treatments. The findings reveal that ripening is characterized by a decline in phenolic content and antioxidant capacity, coupled with an increase in anthocyanin accumulation and metabolic trade-offs that balance antioxidant defenses with pigmentation and sweetness development. Thermal analysis of cell wall components demonstrated a progressive softening process during ripening, highlighting the role of cell wall integrity in fruit texture and postharvest quality.

Importantly, MeJA treatments significantly enhanced enzymatic antioxidant activity (APX, CAT, POD, and SOD) and overall antioxidant capacity (DPPH and FRAP), with repeated applications (T2) eliciting the strongest responses. These treatments not only improved the nutritional profile of the fruit but also reinforced its resilience to oxidative stress, suggesting their potential as a practical tool in horticultural practices. Furthermore, the observed modulation of cell wall stability by MeJA highlights its role in delaying softening, thereby improving fruit firmness and extending shelf life.

The study underscores the dual role of MeJA in enhancing both the functional and marketable quality of blueberries. By modulating antioxidant enzyme activities and secondary metabolite biosynthesis, MeJA not only improves the fruit’s nutritional value but also its resistance to oxidative stress, which is crucial for postharvest management. The findings suggest that MeJA can be integrated into sustainable horticultural practices to meet consumer demand for high-quality, functional fruits. However, the study also highlights the importance of considering environmental factors, such as seasonal variations in temperature and rainfall, which can influence the efficacy of MeJA treatments. This variability underscores the need for tailored application protocols that account for genotype-environment interactions.

While this study provides valuable insights into the effects of MeJA on blueberry ripening and quality, several limitations should be acknowledged. First, the research was conducted over two seasons, and while this provides some insight into interannual variability, longer-term studies are needed to fully understand the environmental impacts on MeJA efficacy. Future research should explore the molecular pathways involved in MeJA’s effects, including the role of specific genes and transcription factors, to optimize application protocols for different cultivars and growing conditions.

## Data Availability

The original contributions presented in the study are included in the article/**Supplementary Material**. Further inquiries can be directed to the corresponding author/s.

## References

[B1] Andler R. Rojas V. Pino V. Castro R. I. Valdés C. Kumar V.. (2023). Efficient production of a polyhydroxyalkanoate by *Azotobacter vinelandii* OP using apple residues as promising feedstock. Int. J. Biol. Macromol. 242, 124626. doi: 10.1016/j.ijbiomac.2023.124626 37119884

[B2] Asghari M. Hasanlooe A. R. (2015). Interaction effects of salicylic acid and methyl jasmonate on total antioxidant content, catalase and peroxidase enzymes activity in “Sabrosa” strawberry fruit during storage. Scientia Hortic. 197, 490–495. doi: 10.1016/j.scienta.2015.10.009

[B3] Asghari M. Hasanlooe A. R. (2016). Methyl jasmonate effectively enhanced some defense enzymes activity and total antioxidant content in harvested “Sabrosa” strawberry fruit. Food Sci. Nutr. 4, 377–383. doi: 10.1002/fsn3.2016.4.issue-3 27247768 PMC4867758

[B4] Balbontín C. Reyes M. Yáñez M. A. Parra-Palma C. Morales-Quintana L. Ramos P. (2025). Enhancing blueberry drought resilience: ABA and MeJA hormonal formulations unveil water-saving strategies. Hortic. Environ. Biotechnol. 66, 99–110. doi: 10.1007/s13580-024-00640-4

[B5] Bari R. Jones J. D. G. (2009). Role of plant hormones in plant defence responses. Plant Mol. Biol. 69, 473–488. doi: 10.1007/s11103-008-9435-0 19083153

[B6] Bujor O.-C. Le Bourvellec C. Volf I. Popa V. I. Dufour C. (2016). Seasonal variations of the phenolic constituents in bilberry (Vaccinium myrtillus L.) leaves, stems and fruits, and their antioxidant activity. Food Chem. 213, 58–68. doi: 10.1016/j.foodchem.2016.06.042 27451155

[B7] Cai H. Han S. Yu M. Ma R. Yu Z. (2020). The alleviation of methyl jasmonate on loss of aroma lactones correlated with ethylene biosynthesis in peaches. J. Food Sci. 85, 2389–2397. doi: 10.1111/1750-3841.15339 32671852

[B8] Castro R. I. Gonzalez-Feliu A. Valenzuela-Riffo F. Parra-Palma C. Morales-Quintana L. (2021a). Changes in the cell wall components produced by exogenous abscisic acid treatment in strawberry fruit. Cellulose. 28, 1555–1570. doi: 10.1007/s10570-020-03607-7

[B9] Castro R. I. Morales-Quintana L. (2019). Study of the cell wall components produced during different ripening stages through thermogravimetric analysis. Cellulose 26, 3009–3020. doi: 10.1007/s10570-019-02305-3

[B10] Castro R. I. Muñoz-Vera M. Morales-Quintana L. (2021b). Evaluation of cell wall modification in two strawberry cultivars with contrasted softness. Agronomy 11, 1100. doi: 10.3390/agronomy11061100

[B11] Castro R. I. Vásquez-Rojas C. Cortiella M. G. I. Parra-Palma C. Ramos P. Morales-Quintana L. (2023). Evolution of the Volatile Organic Compounds, Phenols and Antioxidant Capacity during Fruit Ripening and Development of Rubus ulmifolius Schott Fruits. Horticulturae 9, 13. doi: 10.3390/horticulturae9010013

[B12] Chang C. C. Yang M. Y. Wen H. M. Chern J. C. (2002). Estimation of total flavonoid content in propolis by two complementary colorimetric methods. J. Food Drug Anal. 10, 178–182. doi: 10.38212/2224-6614.2748

[B13] Cheel J. Theoduloz C. Rodríguez J. A. Caligari P. D. S. Schmeda-Hirschmann G. (2007). Free radical scavenging activity and phenolic content in achenes and thalamus from Fragaria chiloensis ssp. chiloensis, *F. vesca* and *F. x ananassa* cv. Chandler. Food Chem. 102, 36–44. doi: 10.1016/j.foodchem.2006.04.036

[B14] Cocetta G. Rossoni M. Gardana C. Mignani I. Ferrante A. Spinardi A. (2015). Methyl jasmonate affects phenolic metabolism and gene expression in blueberry (*Vaccinium corymbosum*). Physiol. Plant 153, 269–283. doi: 10.1111/ppl.12243 24943920

[B15] Delgado L. D. Zúñiga P. E. Figueroa N. E. Pastene E. Escobar-Sepúlveda H. F. Figueroa P. M.. (2018). Application of a JA-ile biosynthesis inhibitor to methyl jasmonate-treated strawberry fruit induces upregulation of specific MBW complex-related genes and accumulation of proanthocyanidins. Molecules 23, 1433. doi: 10.3390/molecules23061433 29899259 PMC6100305

[B16] Erdogan Ü Çakmakçi R. Varmazyari A. Turan M. Erdogan Y. Kitir N. (2016). Role of inoculation with multi-trait rhizobacteria on strawberries under water deficit stress. Agriculture 103, 67–76. doi: 10.13080/z-a.2016.103.009

[B17] Fernández-Calvo P. Chini A. Fernández-Barbero G. Chico J. M. Gimenez-Ibanez S. Geerinck J.. (2011). The Arabidopsis bHLH transcription factors MYC3 and MYC4 are targets of JAZ repressors and act additively with MYC2 in the activation of jasmonate responses. Plant Cell 23, 701–715. doi: 10.1105/tpc.110.080788 21335373 PMC3077776

[B18] Flores G. Blanch G. P. Ruiz del Castillo M. L. (2017). Effect of postharvest methyl jasmonate treatment on fatty acid composition and phenolic acid content in olive fruits during storage. J. Sci. Food Agric. 97, 2767–2772. doi: 10.1002/jsfa.2017.97.issue-9 27754549

[B19] Ghaffari A. Navaee K. Oskoui M. Bayati K. Rafiee-Tehrani M. (2007). Preparation and characterization of free mixed-film of pectin/chitosan/Eudragit RS intended for sigmoidal drug delivery. Eur. J. Pharm. Biopharm 67, 175–186. doi: 10.1016/j.ejpb.2007.01.013 17346954

[B20] Guo X. Y. Zhang X. S. Huang Z. Y. (2010). Drought tolerance in three hybrid poplar clones submitted to different watering regimes. J. Plant Ecol. 3, 79–87. doi: 10.1093/jpe/rtq007

[B21] Gupta V. Estrada A. D. Blakley I. Reid R. Patel K. Meyer M. D.. (2015). RNA-Seq analysis and annotation of a draft blueberry genome assembly identifies candidate genes involved in fruit ripening, biosynthesis of bioactive compounds, and stage-specific alternative splicing. GigaScience 4, 5. doi: 10.1186/s13742-015-0046-9 25830017 PMC4379747

[B22] Huang X. Li J. Shang H. Meng X. (2015). Effect of methyl jasmonate on the anthocyanin content and antioxidant activity of blueberries during cold storage. J. Sci. Food Agric. 95, 337–343. doi: 10.1002/jsfa.6725 24799161

[B23] Huang W. Yao L. He X. Wang L. Li M. Yang Y.. (2018). Hypoglycemic activity and constituents analysis of blueberry (Vaccinium corymbosum) fruit extracts. Diabetes Metab. Syndr. Obes. Targets Ther. 11, 357. doi: 10.2147/DMSO.S166728 PMC6054273 30046248

[B24] Jara K. Castro R. I. Ramos P. Parra-Palma C. Valenzuela-Riffo F. Morales-Quintana L. (2019). Molecular Insights into FaEG1, a Strawberry Endoglucanase Enzyme Expressed during Strawberry Fruit Ripening. Plants 8, 140. doi: 10.3390/plants8060140 31141938 PMC6631567

[B25] Kalt W. Cassidy A. Howard L. R. Krikorian R. Stull A. J. Tremblay F.. (2020). Recent research on the health benefits of blueberries and their anthocyanins. Adv. Nutr. 11, 224. doi: 10.1093/advances/nmz065 31329250 PMC7442370

[B26] Khoo H. E. Azlan A. Tang S. T. Lim S. M. (2017). Anthocyanidins and anthocyanins: colored pigments as food, pharmaceutical ingredients, and the potential health benefits. Food Nutr. Res. 61, 1361779. doi: 10.1080/16546628.2017 28970777 PMC5613902

[B27] Li H. Suo J. Han Y. Liang C. Jin M. Zhang Z.. (2017). The effect of 1- methylcyclopropene, methyl jasmonate and methyl salicylate on lignin accumulation and gene expression in postharvest ‘Xuxiang’ kiwifruit during cold storage. Postharvest Biol. Technol. 124, 107–118. doi: 10.1016/j.postharvbio.2016.10.003

[B28] Li D.-P. Xu Y.-F. Sun L.-P. Liu L.-X. Hu X.-L. Li D.-Q.. (2006). Salicylic acid, ethephon, and methyl jasmonate enhance ester regeneration in 1-MCP-treated apple fruit after long-term cold storage. J. Agric. Food Chem. 54, 3887–3895. doi: 10.1021/jf060240j 16719511

[B29] Li B. Zhang X. Han C. Duan R. Yang J. Xue H. (2023). Effects of methyl jasmonate on fruit coloration and quality improvement in pears (*Pyrus bretschneideri*). Agronomy. 13, 2409. doi: 10.3390/agronomy13092409

[B30] Lohachoompol V. Srzednicki G. Craske J. (2004). The change of total anthocyanins in blueberries and their antioxidant effect after drying and freezing. BioMed. Res. Int. 2004, 485873. doi: 10.1155/S1110724304406123 PMC1082901 15577185

[B31] Morales-Quintana L. Moya M. Santelices-Moya R. Cabrera-Ariza A. Rabert C. Pollmann S.. (2022a). Improvement in the physiological and biochemical performance of strawberries under drought stress through symbiosis with Antarctic fungal endophytes. Front. Microbiol. 13. doi: 10.3389/fmicb.2022.939955 PMC9453553 36090118

[B32] Morales-Quintana L. Tapia-Valdebenito D. Castro R. I. Rabert C. Larama G. Gutiérrez A.. (2022b). Characterization of the Cell Wall Component through Thermogravimetric Analysis and Its Relationship with an Expansin-like Protein in Deschampsia Antarctica. Int. J. Mol. Sci. 23, 5741. doi: 10.3390/ijms23105741 35628551 PMC9143908

[B33] Mustafa M. A. Ali A. Seymour G. Tucker G. (2018). Treatment of dragonfruit (Hylocereus polyrhizus) with salicylic acid and methyl jasmonate improves postharvest physico-chemical properties and antioxidant activity during cold storage. Scientia Hortic. 231, 89–96. doi: 10.1016/j.scienta.2017.09.041

[B34] Neto C. C. (2007). Cranberry and blueberry: evidence for protective effects against cancer and vascular diseases. Mol. Nutr. Food Res. 51, 652–664. doi: 10.1002/mnfr.200600279 17533651

[B35] Parra-Palma C. Morales-Quintana L. Ramos P. (2020). Phenolic content, color development, and pigment related gene expression: A comparative analysis in different cultivars of strawberry during the ripening process. Agronomy 10, 588. doi: 10.3390/agronomy10040588

[B36] Parra-Palma C. Ramos P. Morales-Quintana L. (2025). Optimization of ultrasound-assisted extraction (UAE) of phenolics from blueberries by response surface methodology (RSM). Analytical Lett. 204, 1–19. doi: 10.1080/00032719.2025.2453592

[B37] Parra-Palma C. Valdes C. Muñoz-Vera M. Morales-Quintana L. Castro R. I. (2024). Assessing the modifications and degradation of cell wall polymers during the ripening process of Rubus ulmifolius Schott fruit. J. Hortic. Sci. Biotechnol. 99, 471–479. doi: 10.1080/14620316.2024.2302515

[B38] Silva S. Costa E. M. Veiga M. Morais R. M. Calhau C. Pintado M. (2020). Health promoting properties of blueberries: a review. Crit. Rev. Food Sci. Nutr. 60, 181–200. doi: 10.1080/10408398.2018.1518895 30373383

[B39] Skrovankova S. Sumczynski D. Mlcek J. Jurikova T. Sochor J. (2015). Bioactive compounds and antioxidant activity in different types of berries. Int. J. Mol. Sci. 16, 24673–24706. doi: 10.3390/ijms161024673 26501271 PMC4632771

[B40] Wang H. Kou X. Wu C. Fan G. Li T. (2020). Methyl jasmonate induces the resistance of postharvest blueberry to gray mold caused by Botrytis cinerea. J. Sci. Food Agric. 100, 4272–4281. doi: 10.1002/jsfa.10469 32378217

[B41] Wang Y.-W. Malladi A. Doyle J. W. Scherm H. Nambeesan S. U. (2018). The effect of ethephon, abscisic acid, and methyl jasmonate on fruit ripening in rabbiteye blueberry (Vaccinium virgatum). Horticulturae 4, 24. doi: 10.3390/horticulturae4030024

[B42] Wang H. Wu Y. Yu R. Wu C. Fan G. Li T. (2019). Effects of postharvest application of methyl jasmonate on physicochemical characteristics and antioxidant system of the blueberry fruit. Scientia Hortic. 258, 108785. doi: 10.1016/j.scienta.2019.108785

[B43] Wasternack C. (2007). Jasmonates: an update on biosynthesis, signal transduction and action in plant stress response, growth and development. Ann. Bot. 100, 681–697. doi: 10.1093/aob/mcm079 17513307 PMC2749622

[B44] Wasternack C. Hause B. (2013). Jasmonates: biosynthesis, perception, signal transduction and action in plant stress response, growth and development. Ann. Bot. 111, 1021–1058. doi: 10.1093/aob/mct067 23558912 PMC3662512

[B45] Wu H. Wu X. Li Z. Duan L. Zhang M. (2012). Physiological evaluation of drought stress tolerance and recovery in cauliflower (*Brassica oleracea* L.) seedlings treated with methyl jasmonate and coronatine. J. Plant Growth Regul. 31, 113–123. doi: 10.1007/s00344-011-9224-x

[B46] Xiao B. Sun X. Sun R. (2001). Chemical, structural, and thermal characterizations of alkali-soluble lignins and hemicelluloses, and cellulose from maize stems, rye straw, and rice straw. Polym Degrad Stab 74, 307–319. doi: 10.1016/S0141-3910(01)00163-X

[B47] Zafra-Stone S. Yasmin T. Bagchi M. Chatterjee A. Vinson J. A. Bagchi D. (2007). Berry anthocyanins as novel antioxidants in human health and disease prevention. Mol. Nutr. Food Res. 51, 675–683. doi: 10.1002/mnfr.200700002 17533652

[B48] Zoratti L. Karppinen K. Luengo Escobar A. Häggman H. Jaakola L. (2014). Light-controlled flavonoid biosynthesis in fruits. Front. Plant Sci. 5. doi: 10.3389/fpls.2014.00534 PMC4191440 25346743

[B49] Zuñiga P. E. Castañeda Y. Arrey-Salas O. Fuentes L. Aburto F. Figueroa C. R. (2020). Methyl Jasmonate Applications From Flowering to Ripe Fruit Stages of Strawberry (*Fragaria × ananassa* ‘Camarosa’) Reinforce the Fruit Antioxidant Response at Post-harvest. Front. Plant Sci. 11. doi: 10.3389/fpls.2020.00538 PMC7225341 32457779

