# Supplementary material for: Impact of methyl Jasmonate on blueberry ripening fruits: assessment of cell wall thermal stability, nutritional parameters and antioxidant enzymatic activity
Source: Front Plant Sci. 2025 Mar 27;16:1550131. doi: 10.3389/fpls.2025.1550131 (PMC11983540; doi:10.3389/fpls.2025.1550131)
Supplement: Supplementary file 1 [file Table1.docx]

**Table S1. Criterion for determining color change** (Castro et al. 2022).

| **Parameter** | **Description** |
| --- | --- |
| **ΔE < 0.2** | **Invisibles changes** |
| **0.2 < ΔE < 2** | **Small changes** |
| **2 < ΔE < 3** | **Color changes visible by high quality filter** |
| **3 < ΔE < 6** | **Color changes visible medium quality filter** |
| **6 < ΔE < 12** | **Distinct color changes** |
| **ΔE > 12** | **A different color** |
