# Supplementary figures and images for: Impact of methyl Jasmonate on blueberry ripening fruits: assessment of cell wall thermal stability, nutritional parameters and antioxidant enzymatic activity
Source: Front Plant Sci. 2025 Mar 27;16:1550131. doi: 10.3389/fpls.2025.1550131 (PMC11983540; doi:10.3389/fpls.2025.1550131)

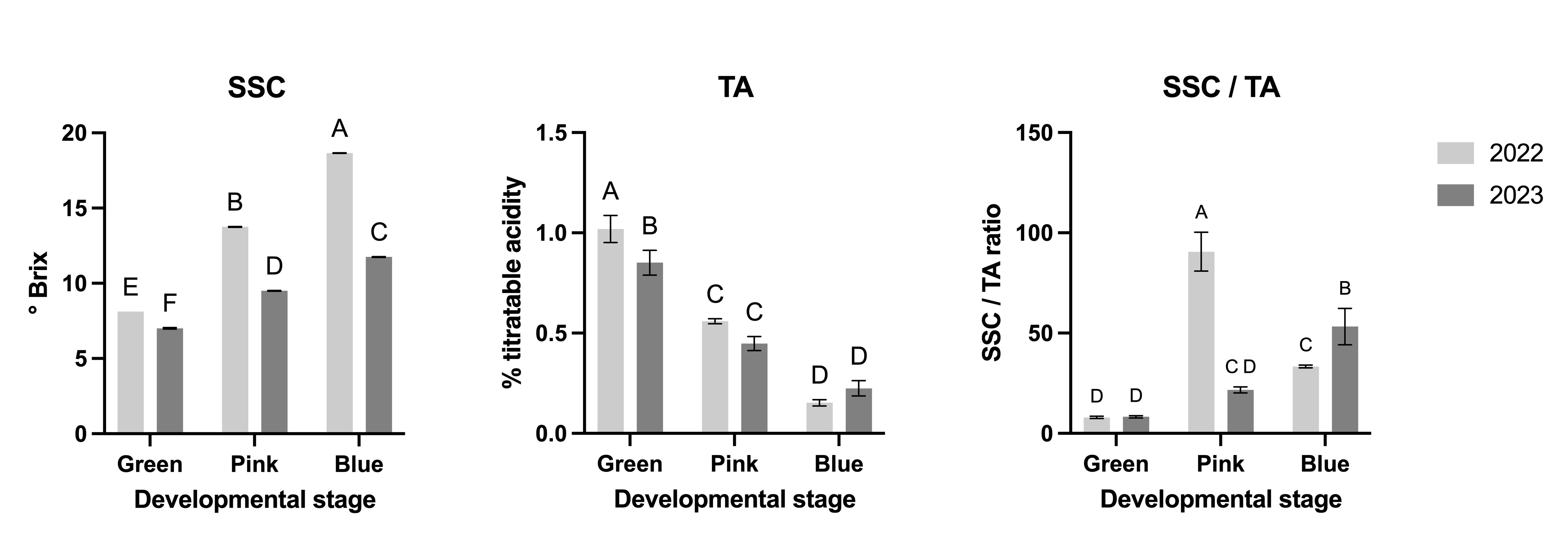

Supplement: Supplementary file 2 [file Image1.jpeg]
